# New Light on the Mind’s Eye: The Pupillary Light Response as Active Vision

**DOI:** 10.1177/0963721415593725

**Published:** 2015-10

**Authors:** Sebastiaan Mathôt, Stefan Van der Stigchel

**Affiliations:** 1 Laboratoire de Psychologie Cognitive, CNRS, Aix-Marseille University, Marseille, France; 2 Department of Experimental Psychology, Helmholtz Institute, Utrecht University

**Keywords:** pupil size, eye movements, visual perception, arousal

## Abstract

The eye’s pupils constrict (shrink) in brightness and dilate (expand) in darkness. The pupillary light response was historically considered a low-level reflex without any cognitive component. Here, we review recent studies that have dramatically changed this view: The light response depends not only on a stimulus’s brightness but also on whether you are aware of the stimulus, whether you are paying attention to it, and even whether you are thinking about it. We highlight the link between the pupillary light response and eye-movement preparation: When you intend to look at a bright stimulus, a pupillary constriction is prepared along with the eye movement before the eyes set in motion. This preparation allows the pupil to rapidly change its size as your eyes move from bright to dark objects and back again. We discuss the implications of these recent advances for our understanding of the subtle yet important role that pupillary responses play in vision.

The eyes’ pupils constrict (shrink) in brightness and dilate (expand) in darkness. This is the *pupillary light response*. The light response has been studied for more than a millennium (Loewenfeld, 1958) but was historically considered a low-level reflex without any cognitive component. However, recent studies have shown that the light response is far more than a reflex, and reveals what you attend to, how you interpret what you see, and even what you think about. Here, we review these recent advances in our understanding of the pupillary light response. In addition, we discuss how changes in pupil size help to find the optimal balance between visual acuity (how sharp you can see) and sensitivity (how well you can detect faint stimuli) and are therefore a crucial aspect of how you perceive the world.

## The Light Response Reflects Awareness, Interpretation, and Mental Imagery

**Fig. 1.**
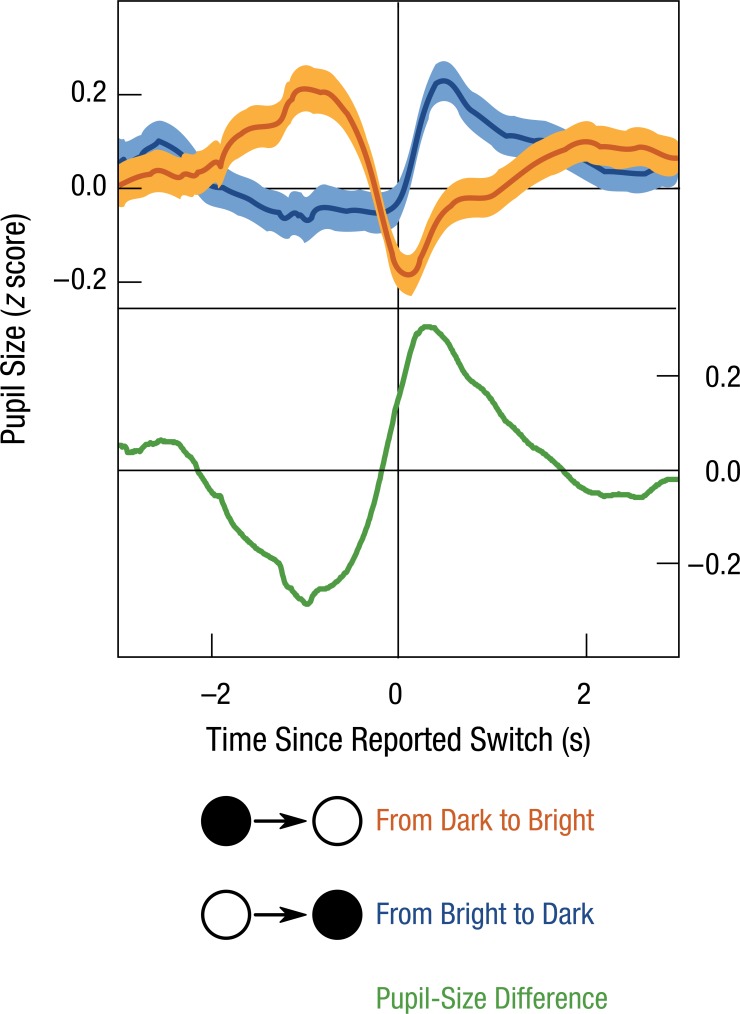
The effect of visual awareness on the pupillary light response in a binocular rivalry experiment (Naber, Frassle, & Einhauser, 2011). When awareness switches from a dark stimulus (presented to one eye) to a bright stimulus (presented to the other eye), the pupil constricts (orange line). Conversely, when awareness switches from a bright to a dark stimulus, the pupil dilates (blue line). On the *x* axis, 0 seconds corresponds to the moment that the participant indicates that his or her awareness has switched. Error bands indicate standard errors.

Cognitive effects on the light response were first shown using binocular rivalry (e.g., Harms, 1937; Naber, Frassle, & Einhauser, 2011). In binocular rivalry, different stimuli are presented to each eye. When the stimuli are too different to be fused into a single percept, visual awareness flips back and forth between the left and right eye. (You can experience binocular rivalry by looking at your own nose. Although each eye sees your nose from a different angle, you consciously perceive your nose from only one angle.) With respect to the light response, something remarkable happens when images of different brightness are presented to each eye: The pupil constricts when the bright stimulus, relative to the dark stimulus, dominates awareness (see Fig. 1). The light response therefore reflects which stimulus you consciously perceive at a given moment.

Similarly, recent studies have shown that the pupil responds to the perceived brightness of pictures, which is not always the same as their actual brightness (Binda, Pereverzeva, & Murray, 2013b; Laeng & Endestad, 2012; Naber & Nakayama, 2013). For example, a picture of a sun is generally perceived as brighter, and elicits a stronger pupillary constriction, than a picture of an indoor scene—even when both pictures are really equally bright. Strikingly, your pupil even constricts when you imagine a bright stimulus, without any visual stimulation (Laeng & Sulutvedt, 2014). Together, these studies show that the light response is not driven solely by the amount of light that enters the eye but is related to high-level vision and even mental imagery.

## The Light Response Reflects Visual Attention

You always see multiple objects. While you work on your computer, you look at your screen but, from the corner of your eye, might also see your keyboard and cup of coffee. You do not fully process everything you see, but selectively attend to only a few objects at a time. If you attend to an object, you respond to it more quickly, and perceive it more clearly (Carrasco, 2011). A crucial question is whether attention affects vision even at the earliest possible stage: as light enters the eye through the pupil.

**Fig. 2.**
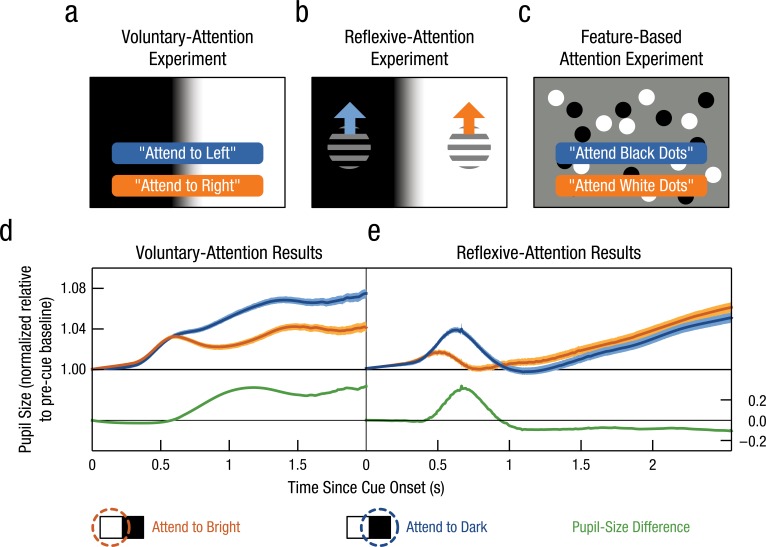
The effect of covert visual attention on the pupillary light response. Panel (a) shows an example of a voluntary-attention experiment in which participants direct their attention to the left or right side of the screen based on an auditory cue (cf. Mathôt, van der Linden, Grainger, & Vitu, 2013). Panel (b) shows an example of a reflexive-attention experiment in which attention is drawn to the left or right by a sudden movement (cf. Mathôt, Dalmaijer, Grainger, & Van der Stigchel, 2014). Panel (c) shows an example of a feature-based-attention experiment in which participants attend to one of two intermingled sets of dots (cf. Binda et al., 2014b). The pupil is larger when attention is voluntarily directed at a dark (blue), relative to a bright (orange), surface (d; Mathôt et al., 2013). After a reflexive shift of attention, the pattern is initially similar (i.e., a larger pupil when attending to a dark surface) but inverses after about 1 second, corresponding to inhibition of return (e; Mathôt et al., 2014). Error bands correspond to 95% confidence intervals.

The effect of attention on the pupillary light response was recently tested in several studies (Binda, Pereverzeva, & Murray, 2013a; Mathôt, van der Linden, Grainger, & Vitu, 2013; Naber, Alvarez, & Nakayama, 2013). In one of our experiments, participants looked at the center of a display that was divided into a bright and a dark half (Fig. 2a; Mathôt et al., 2013). Participants identified a target stimulus that could appear on either side of the screen, on the bright or the dark background. We used a staircase procedure to ensure that there was no difference in how easily the target could be identified on a dark or a bright background. Just before the target stimulus appeared, a cue (a voice saying “left” or “right”) indicated the probable location of the target. Participants used this information to anticipate the location of the target and shifted their attention to the cued side of the screen while keeping their eyes on the display center (i.e., covert attention).

Figure 2d shows the results of this experiment. First, overall pupil size increased over time, regardless of the brightness of the attended side. This was related to the effort that participants invested in the task, which affects pupil size in a way that is more or less independent of the light response (reviewed in Beatty, 1982; Goldwater, 1972; Laeng, Sirois, & Gredebäck, 2012). More importantly, when participants attended to the bright side of the screen (orange line), their pupils constricted relative to when they attended to the dark side (blue line). This difference arose about 0.6 seconds after the cue was presented.

This experiment showed that your pupils adjust to an object if you attend to it, even if you do not look at it directly. This is important, because elements in a visual scene can differ strongly in brightness: Your keyboard might be dark, whereas your monitor might be bright. While looking at your monitor, you may covertly (i.e., without moving your eyes) attend to your keyboard to localize your fingers. As we will describe later in this review, perception benefits from an optimal pupil size, so if you attended to the keyboard with a pupil size that was tuned to the brightness of the monitor, this would result in suboptimal perception. Therefore, even though the benefit is presumably small, a link between the pupillary light response and visual attention is beneficial.

In the experiment described above, participants shifted their attention voluntarily. But attention can also be drawn involuntary toward a location. Such reflexive shifts of attention are typically studied by presenting a salient cue, such as a sudden movement, in your visual periphery (Fig. 2b). Even when this cue is irrelevant for the task, it still captures attention.

We recently showed that the pupillary light response is also affected by reflexive shifts of attention in the absence of eye movements (Mathôt, Dalmaijer, Grainger, & Van der Stigchel, 2014). As shown in Figure 2e, participants’ pupils first constricted when the cue was presented on a bright background (orange line), relative to a dark (blue line) background, but relatively dilated for longer intervals after cue presentation (i.e., the pupil-size difference, indicated in green, switched from positive to negative). The early constriction reflects a rapid reflexive shift of attention to the cued location, whereas the later dilation reflects a phenomenon called *inhibition of return*. In a behavioral response-time task, inhibition of return refers to the finding that responses are slower when a target is presented at a cued location, relative to an uncued location, for long intervals between the cue and the target. This is likely a “been there, done that” mechanism that prevents attention from being drawn to the same location over and over again. Interestingly, participants who showed strong pupillary inhibition (i.e., the negative pupil-size difference in Fig. 2e) also showed strong inhibition of return (i.e., slowed responses to targets that appeared on the cued side of the screen).

Attention may be drawn not only to locations but also to features such as color and shape. For instance, while approaching a bookshelf looking for a specific red book, you can attend to the red books only. Binda, Pereverzeva, and Murray (2014) showed that pupil size is also an index of feature-based attention. In their experiment, two sets of dots (one bright, one dark) were presented at the same location (Fig. 2c). Participants could therefore select the cued set of dots only on the basis of its brightness. Crucially, the pupil constricted when the bright dots, relative to the dark dots, were attended. This shows that the pupil is not only an index of spatial attention but a proxy for various forms of selective attention: Whatever visual information is important (be it feature or location) will be echoed by the pupil.

## The Light Response Reflects Eye-Movement Preparation

In the experiments described above, participants did not move their eyes. This is artificial, because in daily life you usually look directly at what you attend to. Therefore, numerous researchers have proposed that attention and eye movements are linked (Rizzolatti, Riggio, Dascola, & Umiltá, 1987): Whenever you shift your attention, you also prepare an eye movement to the attended location. To come back to our daily-life example: When you look at your monitor but want to localize your fingers, you quickly make an eye movement to the keyboard. Before this eye movement is executed, attention has already shifted to the end point of the eye movement. However, the to-be-fixated object (the keyboard) might have a different brightness than the currently fixated object (the screen). An important question is whether the pupil prepares for this change in brightness. We recently showed that this is indeed the case: When you prepare an eye movement toward a bright object, a pupillary constriction is prepared along with the eye movement itself, before the bright object has been fixated (Mathôt, van der Linden, Grainger, & Vitu, 2015). This is useful, because it reduces the effective latency of the light response, which is long (approximately 0.25 s). This way, preparation allows the pupil to track the rapid changes in visual input that occur as your eyes shift from dark to bright objects and back again.

## A Balance Between Visual Acuity and Sensitivity

Although the light response is the primary determinant of pupil size, the pupil also dilates in response to arousal in a way that is independent of the light response. Here, we use “arousal” in its broadest sense, sometimes described as “the intensity dimension of thought” (Just & Carpenter, 1993). In general, anything that increases arousal also elicits a pupillary dilation: sexy pictures, mental arithmetic, keeping something in working memory, effortful listening, etcetera (reviewed in Beatty, 1982; Goldwater, 1972; Laeng et al., 2012). Irene Loewenfeld, one of pupillometry’s pioneers, aptly summarized that “man may either blush or turn pale . . . but his pupils always dilate” (1958, p. 237).

But why does the pupil respond to arousal and light in this way? The same pupillary responses are found across many vertebrate species and have even evolved independently in squids and octopuses (Douglas, Williamson, & Wagner, 2005), strongly suggesting that they serve an important function. Although there is no definite answer, there are several credible hypotheses that each explain one aspect of the pupillary response. Below, we synthesize these hypotheses to provide a comprehensive understanding of the important role that pupillary responses play in vision.

One function of the light response is to find a balance between visual acuity (how sharp you can see) and sensitivity (how well you can detect faint stimuli). The eye’s lens is imperfect and distorts light in ways that reduce acuity. The severity of these distortions depends on pupil size: the smaller the pupil, the sharper the image (Campbell & Gregory, 1960). Another benefit of a small pupil is that it sees sharply across a wide range of distances (i.e., increased depth of field). However, a small pupil also has disadvantages: It does not capture much light, which leads to reduced sensitivity, and provides a slightly reduced field of view. Crucially, the optimal size of the pupil depends on how much light is available. In darkness, vision is limited by the scarcity of light, and the pupil therefore dilates to capture more light. In brightness, light is abundant, and the pupil therefore constricts to obtain the sharpest image. Plausibly, cognitive effects on the pupillary light response may serve to optimize pupil size specifically for objects that you attend to, or prepare an eye movement toward.

But why does arousal trigger a pupillary dilation, apparently perturbing the balance between visual acuity and sensitivity? This may be related to Aston-Jones and Cohen’s (2005) proposal that there are two modes of behavior, exploitation and exploration, that are linked to pupil size. During exploitation, arousal is low (compared to exploration), and you are focused on one task, such as reading a book, that requires fine visual discrimination. In this mode, visual acuity is more important than sensitivity, and the pupil therefore constricts. During exploration, arousal is high, and you are in a vigilant state, ready to detect mates, predators, and other things that require immediate action. In this mode, visual sensitivity is more important than acuity, and the pupil therefore dilates. Pupil dilation in the absence of light changes may thus reflect a shift from exploitation to exploration mode, and a concomitant shift in the optimal balance between visual acuity and sensitivity.

## Taken Together . . .

. . . it is clear that the pupillary light response is far more than the low-level reflex that it was historically thought to be. The extent to which a bright stimulus triggers a pupillary constriction depends on many cognitive factors: visual awareness (are you consciously aware of the stimulus?), interpretation (how bright does the stimulus subjectively appear?), and visual attention (are you paying attention to the stimulus?). We have emphasized the link between pupillary responses and eye-movement preparation: When you prepare to look at a bright stimulus, a pupillary constriction is prepared along with the eye movement itself. Preparation allows the pupil to rapidly adjust its size, as your eyes shift from dark to bright objects and back again.

We have highlighted the important role that pupillary responses play in vision. The pupillary response to light balances visual sensitivity, which is highest for large pupils, and acuity, which is highest for small pupils (Campbell & Gregory, 1960; see Woodhouse & Campbell, 1975, for other functions). The function of pupillary dilation in response to arousal is less clear but may be understood in the same way: Arousing situations are generally those that require enhanced visual sensitivity, and the pupil therefore dilates when aroused. In summary, the pupillary light response reflects mental state in exquisite detail. It is truly a mind’s eye.
